# Annotations

**Published:** 1895-03-30

**Authors:** 


### ANNOTATIONS.

### Should First Cousins Marry?

THE other day several members of a family, who were either deaf, or dumb, or both, came within the writer's cognizance, and it was ascertained that they were all the offspring of first cousins. It has long been an established maxim that first cousins ought not to marry, though the rule is very often broken. The question of the actual anatomical and physiological causes of physical disabilities in the offspring of first cousins is well worthy of the most thorough investigation. In a complex vital organism like the human body, one need not be surprised to find occasional or even frequent departures from the normal standard of physical and mental perfection. Such departures will naturally vary in kind and degree in different families. Thus, one family may have defective lungs, another feeble hearts, another inactive livers, another poor eyesight, another an inadequate auditory apparatus, and so on. If the members of such families are fortunate enough to marry persons who are free from the same class of anatomical and physiological peculiarities, such peculiarities will naturally tend to be diminished, perhaps by so much as half, in their offspring, and in the course of generations of physiologically fortunate marriages they may disappear. But if, on the other hand, persons of the same blood and family, such as first cousins, all of whom must necessarily be more or less similar in structure and function, marry each other, then their peculiarities will tend, not to be diminished, but to be increased, perhaps doubled. So that, taking hearing as a sense which is somewhat deficient in a given family, one would expect that two first cousins marrying, whose hearing tended to be imperfect, would produce offspring who would be very likely to be deaf, and that not merely in old age, but in youth, or even in childhood. The same, of course, is true of all sorts of physical and mental peculiarities as well as of hearing. If people would but bear in mind that the union of persons who have similar defects, more especially if they are blood relations, tends to intensify those defects, exactly as piling coal on a bright fire infallibly produces augmented heat, they would save themselves much bitter remorse, and avoid the maledictions of a class of imperfect human beings who not seldom curse the day they were born.

### Spring Headaches.

IT is not sufficiently considered to what an extent, all over the world, health is a matter of climate and seasonal change. During the past two or three weeks, as is usual at this period of the year, there has been a great prevalence of headaches, accompanied by depression of spirits and inability, and even unwillingness to work. More especially has this been the case with those whose work is indoors—in offices, in studies, in shops, and in work-rooms. The causes of these headaches are not always obvious to those who suffer from them, though a little careful and continuous search often brings them to light, and then they are seen to be, at any rate partially, under our own control. Now, to discover and remove the causes of ills is to do better than to cure them; it is to prevent them. One very common cause of headaches in large numbers of persons during the recent season was the sudden change from the dry, frosty, bracing atmosphere which had prevailed for several weeks to a warm, moist, relaxing atmospheric condition. The change relaxed all the blood vessels of the body, and thus withdrew a certain quantity of blood from the nerve centres, with the result of lowering nerve nutrition and producing nerve and brain debility, with lassitude and headache. Another common cause just now is the hot rooms in which so many people still indulge. In March it would, of course, be absurd to think of giving up fires; but a very careful regulation of them, so as to produce a uniform and strictly moderate temperature indoors, is very greatly to be desired. Our outdoor climate we cannot well regulate, though by intelligent and sufficiently frequent changes of clothing we can adapt ourselves to it. But our indoor climate we can regulate, and thus prevent many a debilitated brain from sinking down to the neurasthenic level which a headache reveals. In truth, a few visits to the doctor, with tonic treatment and a brief but bracing change of air for all of us who dwell in towns, are what nature and reason alike indicate at the present season.

### The Religious Question at the Tottenham Hospital.

THE dispute between the medical staff of the Tottenham Hospital and the council of the institution has had its origin in the religious question. The hospital, it may be remembered, is only part of a large institution, an institution—we quote from the trust deed—which has for its object "the training of Christian women to serve as deaconesses, that is to say, as working, teaching, and nursing sisters, but who shall not be subject to any obligation or vow of celibacy. A training hospital shall always be maintained as part of the institution." In endeavouring to judge rightly of the merits of the present disagreement, it is to be borne in mind that the primary object of the institution is "the training of Christian women as deaconesses." The hospital is used, not as a main object in itself, but only as a means to an end. But the institution is under this primary Christian obligation to the patients and the medical staff—to do to them exactly as it would be done by. No one can doubt that a sick person who entrusts himself to the wards of the hospital wishes, in the first place and above all things else, to be cured of his disease. It is equally clear that the doctor or surgeon who has charge of the patient wishes, first and before all things else, to cure his patient of his disease. From this it follows that it is the duty of the institution to give the doctor and the patient each an entirely free hand so long as there is disease or danger present. In other words, the doctor, and the doctor alone, must give all instructions which concern the patient's well-being, and those instructions must be loyally obeyed by nurses and all other persons concerned. When the doctor says that a patient may with safety be made the subject of religious ministrations, then, and not until then, may those ministrations be commenced. All this is manifest to plain sense. It is to be feared that the well-meaning, but, perhaps, a little over-zealous, deaconesses at Tottenham have not been sufficiently mindful of the elementary principles of hospital administration. We sincerely hope that a word in season may induce them to reconsider the situation. The question demands their best wisdom for many and grave reasons.

